# Optimizing double-layered convolutional neural networks for efficient lung cancer classification through hyperparameter optimization and advanced image pre-processing techniques

**DOI:** 10.1186/s12911-024-02553-9

**Published:** 2024-05-27

**Authors:** M. Mohamed Musthafa, I. Manimozhi, T. R. Mahesh, Suresh Guluwadi

**Affiliations:** 1 Al-Ameen Engineering College (Autonomous), Erode, Tamil Nadu India; 2 Department of Computer science and Engineering, East Point College of Engineering & Technology, Bangalore, India; 3 Department of Computer Science and Engineering, JAIN (Deemed-to-be University), Bengaluru, 562112 India; 4 Adama Science and Technology University, Adama, 302120 Ethiopia

**Keywords:** Lung cancer, Machine learning, CNN, SMOTE, Image preprocessing, CT scans, Classification, Diagnostic accuracy

## Abstract

Lung cancer remains a leading cause of cancer-related mortality globally, with prognosis significantly dependent on early-stage detection. Traditional diagnostic methods, though effective, often face challenges regarding accuracy, early detection, and scalability, being invasive, time-consuming, and prone to ambiguous interpretations. This study proposes an advanced machine learning model designed to enhance lung cancer stage classification using CT scan images, aiming to overcome these limitations by offering a faster, non-invasive, and reliable diagnostic tool. Utilizing the IQ-OTHNCCD lung cancer dataset, comprising CT scans from various stages of lung cancer and healthy individuals, we performed extensive preprocessing including resizing, normalization, and Gaussian blurring. A Convolutional Neural Network (CNN) was then trained on this preprocessed data, and class imbalance was addressed using Synthetic Minority Over-sampling Technique (SMOTE). The model’s performance was evaluated through metrics such as accuracy, precision, recall, F1-score, and ROC curve analysis. The results demonstrated a classification accuracy of 99.64%, with precision, recall, and F1-score values exceeding 98% across all categories. SMOTE significantly enhanced the model’s ability to classify underrepresented classes, contributing to the robustness of the diagnostic tool. These findings underscore the potential of machine learning in transforming lung cancer diagnostics, providing high accuracy in stage classification, which could facilitate early detection and tailored treatment strategies, ultimately improving patient outcomes.

## Introduction

Lung cancer stands as a formidable global health challenge, consistently ranking as one of the leading causes of cancer-related mortality worldwide. It is characterized by the uncontrolled growth of abnormal cells in one or both lungs, typically in the cells lining the air passages. Unlike normal cells, these cancerous cells do not develop into healthy lung tissue; instead, they divide rapidly and form tumors that disrupt the lung’s primary function: oxygen exchange.

The global impact of lung cancer is staggering, with millions of new cases diagnosed annually. Its high mortality rate is primarily due to late-stage detection, where the cancer has progressed to an advanced stage or metastasized to other body parts, significantly diminishing the effectiveness of treatment modalities. Thus, early and accurate diagnosis of lung cancer is paramount in improving patient prognoses, extending survival rates, and enhancing the quality of life for affected individuals.

The primary cause of lung cancer is cigarette smoking, which exposes the lungs to carcinogenic substances that can damage the cells’ DNA and lead to cancer. Other risk factors for lung cancer include exposure to secondhand smoke, radon gas, asbestos, air pollution, and a family history of lung cancer.

Symptoms of lung cancer can vary but may include persistent coughing, chest pain, shortness of breath, hoarseness, coughing up blood, unexplained weight loss, and fatigue. However, lung cancer may not cause symptoms in its initial stages, which is why early detection through screening is crucial for improving outcomes.

Diagnosis of lung cancer typically involves imaging tests such as chest X-rays, CT scans, and PET scans to visualize the lungs and detect any abnormalities. A biopsy, where a small sample of lung tissue is taken and examined under a microscope, is usually needed to confirm the diagnosis.

Treatment options for lung cancer depend on several factors, including the type and stage of the cancer, as well as the patient’s overall health and preferences. Treatment may include surgery to remove the tumor, chemotherapy, radiation therapy, targeted therapy, immunotherapy, or a combination of these approaches.

**Fig. 1:**
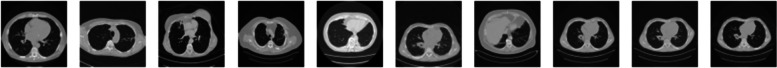
Sample images of lung cancer

Lung cancer is a critical condition that necessitates immediate medical care. Detecting it early, along with improvements in treatment methods, has enhanced the prognosis for numerous patients. However, the most effective strategy to avoid lung cancer is to stop smoking and minimize contact with additional risk elements. Figure 1 displays some example images of lung cancer tests.

Current diagnostic techniques for lung cancer involve various approaches, such as biopsies, CT scans, chest X-rays, PET scans, and MRI, among others [1]. While these methods are invaluable in the diagnostic process, they come with certain limitations. For instance, biopsies, while definitive, are invasive and carry risks of complications. Less invasive imaging methods such as X-rays or CT scans might produce false positives or negatives, potentially causing unwarranted stress or delays in treatment.

Moreover, the interpretation of these diagnostic tests heavily relies on the expertise of the clinician, introducing a degree of subjectivity and potential for human error. There’s also the challenge of early-stage lung cancer, which often presents very subtle changes not always detectable with conventional imaging techniques [2].

This context highlights the critical need for advanced diagnostic tools capable of overcoming these challenges. This study aims to address these issues by developing a machine learning model using Convolutional Neural Networks (CNNs) to enhance the precision and effectiveness of lung cancer stage classification from CT scans. By automating and refining the diagnostic process, the proposed model seeks to mitigate the limitations of traditional methods, offering a faster, non-invasive, and more reliable diagnostic alternative.

The impact of this study is significant: the model’s high accuracy in classifying lung cancer stages promises to revolutionize clinical diagnostics, facilitating early detection and enabling tailored treatment strategies. This advancement has the potential to improve patient outcomes by allowing for timely intervention and more effective management of lung cancer, ultimately contributing to reduced mortality rates and enhanced patient care.

The objective of this research paper is to:

- Develop a machine learning model utilizing Convolutional Neural Networks (CNNs) for lung cancer stage classification based on CT scans.
- Bridge existing diagnostic deficiencies by providing clinicians with a tool for expedited and precise decision-making in lung cancer management.
- Contribute to improved patient outcomes through enhanced diagnostic accuracy and early detection capabilities.

The paper is organized as follows: Initially, the Literature Review explores existing research on lung cancer diagnostics, highlighting advancements and limitations, and sets the foundation for the proposed methodology. Subsequently, the Materials and methods section describes the dataset, preprocessing steps, model architecture, training process, and evaluation metrics in detail. The Results section then presents the study’s findings, including model performance metrics and comparative analysis with existing methods. This is followed by the Discussion, which interprets the results, discusses implications for clinical practice, addresses limitations, and suggests future research directions. Finally, the Conclusion summarizes the main findings and their relevance within the broader scope of lung cancer diagnostics, supported by a comprehensive list of References to provide credit and enable readers to explore the research background further.

Through this structured approach, the paper aims to contribute meaningful insights to the field of medical imaging and machine learning, offering a novel tool for the early and accurate diagnosis of lung cancer.

## Literature review

The literature surrounding lung cancer diagnostics encompasses various methodologies, ranging from traditional imaging techniques to more advanced approaches such as machine learning. This review aims to explore existing research in this area, highlighting both the advancements made and the limitations faced, ultimately setting the foundation for the proposed machine learning-based methodology.

### Diagnosis of lung cancer using CT scans

The utilization of Computed Tomography (CT) scans in lung cancer diagnosis has been a cornerstone in the medical field, offering high-resolution images that are pivotal for detecting and monitoring various stages of lung tumors [3]. Over the years, numerous studies have underscored the importance of CT scans in identifying nodules that could potentially be malignant, with a particular focus on low-dose CT scans, which have become a standard in screening programs, especially for high-risk populations. Such studies underscore the superior sensitivity of CT scans in identifying early-stage lung cancer, a significant advancement over other imaging methods like chest X-rays, which may overlook smaller, subtler lesions.

Despite the advancements, the interpretation of CT scans remains a significant challenge. Radiologists need to discern between benign and malignant nodules, an endeavor complicated by the presence of various artifacts and benign conditions like scars or inflammatory diseases, which can mimic the appearance of cancerous nodules [4, 5].

### Machine learning approaches in lung cancer detection and classification

The integration of machine learning, particularly deep learning techniques, into the analysis of CT images has established a groundbreaking paradigm in the identification and classification of lung cancer. Convolutional Neural Networks (CNNs) are spearheading this transformation by providing a framework for automated extraction and categorization of features directly from the images. This advancement marks a substantial stride in augmenting the accuracy and effectiveness of lung cancer diagnostics, thus facilitating more precise and timely interventions.

#### Binary classification models

Early studies primarily focused on binary classification, distinguishing between malignant and non-malignant nodules. CNNs, through their layered architecture, have demonstrated the ability to learn complex patterns in imaging data, surpassing traditional computer vision techniques in accuracy and reliability [6, 7].

#### Multi-class classification models

Recent advancements have moved towards more nuanced multi-class classification models that categorize nodules into various cancer stages or types. This granularity is crucial for treatment planning and prognosis, offering a more detailed understanding of the disease’s progression [8].

#### Transfer learning

Given the challenges of assembling large annotated medical imaging datasets, transfer learning has become a popular approach. Models pre-trained on vast, non-medical image datasets are fine-tuned on smaller medical imaging datasets, leveraging learned features to improve performance in the medical domain [9].

#### Data augmentation

To address the issue of restricted training data, strategies such as rotation, scaling, and flipping are commonly employed for data augmentation, effectively expanding the training dataset artificially. These methods bolster the model’s resilience and its ability to generalize from a limited number of examples [10].

#### Segmentation models

Deep learning models extend their utility beyond mere classification; they are also employed in segmentation tasks, delineating the precise boundaries of nodules, which is vital for assessing tumor size and growth over time. U-Net, a type of CNN, is particularly noted for its effectiveness in medical image segmentation [11].

In Table 1 a few of the studies which have been done in this field are given.

**Table 1:** Related work

Study	Objective
Marjolein A. Heuvelmans, et al. (2021) [12]	The LCP-CNN demonstrates excellent performance in identifying benign lung nodules in an independent European dataset, with a 95% accuracy.
Nguyen Quoc Khanh Le et al. (2021) [13]	The machine learning-based model predicts EGFR and KRAS mutations in NSCLC patients with accuracies of 83.6% and 86% respectively.
Ying Xie et al. (2024) [14]	The proposed method exhibits significant diagnostic strength for early lung cancer detection, achieving an accuracy of 96.8%.
Zhang Li et al. (2021) [15]	Deep learning methods for lung cancer segmentation achieved an accuracy of 83.98%.
Sanjana Narvekar et al. (2022) [16]	Various machine learning techniques including ANN, SVM, CNN, KNN, and NBC achieved an accuracy of 97.2%.
Mattakoyya Aharonu et al. (2022) [17]	A CNN-based framework achieved an accuracy of 94.11% in lung cancer identification.
B C Kavitha et al. (2022) [18]	Neural networks achieved an accuracy of 94% in lung cancer detection.
Jason L. Causey (2022) [19]	Combination of Spatial Pyramid Pooling and 3D Convolution achieved an accuracy of 89.2% in lung cancer segmentation.
Imran Ahmed et al. (2023) [20]	Deep learning architectures reached accuracies ranging from 93–94% in lung cancer detection.

### Gaps in current research

Despite significant advancements in lung cancer diagnostics, several critical gaps remain in the current research landscape. Many existing models are trained on datasets lacking diversity in demographics, scanner types, and image acquisition parameters, which can limit their generalizability across different populations and clinical settings. This limitation underscores the need for more comprehensive and diverse datasets to enhance the robustness of diagnostic models. Additionally, the “black box” nature of deep learning models poses a challenge for clinical adoption, as there is a growing demand for models that not only predict accurately but also provide insights into the reasoning behind their predictions. This issue of interpretability is crucial for gaining the trust of clinicians and integrating these models into clinical workflows effectively. Furthermore, the transition from research to clinical practice is slow, with models requiring not just technological solutions but also addressing regulatory, ethical, and practical considerations to facilitate their integration into routine medical care. Another critical gap is the need for models capable of longitudinal analysis, which can analyze changes in lung nodules over time, providing a dynamic assessment that aligns more closely with clinical needs. Addressing these gaps, this study introduces a comprehensive CNN model trained on a diverse and extensive dataset, encompassing various stages of lung cancer. The model is designed for multi-class classification, offering detailed insights critical for personalized treatment strategies. Emphasis is placed on the interpretability of the model, aiming to provide clinicians with understandable and actionable information. By demonstrating the model’s effectiveness in a clinical setting, this research contributes to the ongoing effort to integrate advanced machine learning techniques into the realm of lung cancer diagnosis and treatment.

Addressing these gaps, this study introduces a comprehensive CNN model trained on a diverse and extensive dataset, encompassing various stages of lung cancer. The model is designed for multi-class classification, offering detailed insights critical for personalized treatment strategies. Emphasis is placed on the interpretability of the model, aiming to provide clinicians with understandable and actionable information. By demonstrating the model’s effectiveness in a clinical setting, this research contributes to the ongoing effort to integrate advanced machine learning techniques into the realm of lung cancer diagnosis and treatment.

## Materials and methods

**Fig. 2:**
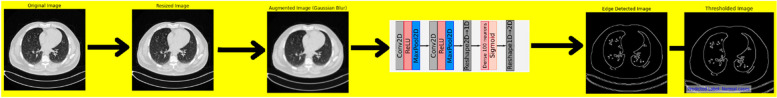
Workflow of the proposed model

This section delineates the comprehensive methodology employed to construct and validate a convolutional neural network (CNN) model for the classification of lung cancer stages using the IQ-OTHNCCD lung cancer dataset. The approach encompasses dataset acquisition, application of preprocessing methodologies, formulation of the model architecture, delineation of training procedures, and determination of evaluation metrics to ensure a comprehensive and reliable analysis. The workflow of the proposed model is visually depicted in Fig. 2.

### Dataset description and preprocessing

The IQ-OTHNCCD lung cancer dataset, integral to this study, is painstakingly curated to facilitate the creation and validation of machine learning models aimed at identifying and classifying lung cancer stages. This dataset encompasses a vast collection of CT scan images essential for advancing diagnostic capabilities in the field of lung cancer.

This dataset comprises CT scan images, comprising a diverse and comprehensive range of cases, covering various stages of lung cancer, including benign, malignant, and normal cases. This diversity is essential for training robust models capable of generalizing well across the spectrum of lung cancer manifestations, enabling effective diagnostic applications. In Table 2 a brief description of the dataset has been given.

**Table 2:** Dataset description

Type	Number of Samples
**Malignant**	561
**Benign**	120
**Normal**	416
**Total**	**1097**

**Fig. 3:**
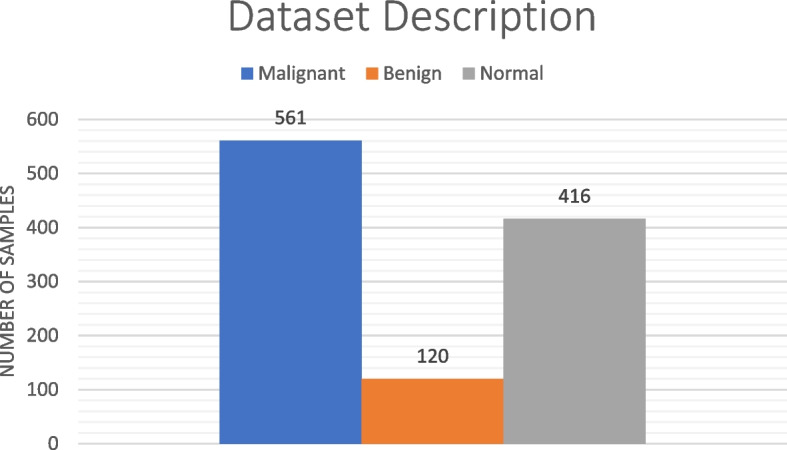
Dataset description

Based on Table 2, to provide visual insights of the data Fig. 3 delves into the same aspects.

Annotating and labeling each image meticulously, medical professionals from the Iraq-Oncology Teaching Hospital/National Center for Cancer Diseases have ensured the dataset’s reliability. Annotations categorize images into one of three classes: benign, malignant, or normal. Such granular labeling establishes a solid ground truth essential for training and assessing the model, enhancing the dataset’s utility in research and clinical applications.

Characterized by high quality and consistency, the CT scans adhere to standardized imaging protocols, guaranteeing reliability and accuracy. However, variations in image dimensions necessitate preprocessing to standardize inputs for neural networks. These steps ensure that the model processes uniform data, enhancing its performance and generalizability across diverse datasets. The images of the dataset ratio are checked using Eq. 1.

1 $$Dataset\;Balance\;Ratio=\frac{\text{Number of }{\text{Samples}}_\text{Majority Class}}{\text{Number of }{\text{Samples}}_\text{Minority Class}}$$

Preprocessing steps are pivotal in preparing data for effective model training, including:

Resizing: Resizing images to a uniform dimension ensures consistency in input size for CNNs, optimizing model performance.

Normalization: Normalizing pixel values to a scale of 0 to 1 expedites model convergence during training, facilitating efficient learning. It is achieved using Eq. 2.

2 $$\text{Pixel Normalization}=\frac{\text{Pixel Value}}{\text{Maximum Pixel Value}}$$

Augmentation: Utilizing data augmentation methods like rotation, flipping, and scaling improves the model’s robustness and helps prevent overfitting by effectively enlarging the dataset size.

Splitting: Partitioning the dataset into training, validation, and test sets is crucial for facilitating effective model training and evaluation, thereby ensuring the model’s ability to generalize and perform accurately on unseen data.

In this process, CNN is trained using the preprocessed dataset to adeptly extract features from CT scan images and accurately classify the stages of lung cancer. The dataset’s diversity and quality are pivotal in enabling the model to learn nuanced features and patterns associated with various lung cancer stages, underscoring its significance in advancing diagnostic accuracy and efficiency.

The IQ-OTHNCCD lung cancer dataset serves as the cornerstone for developing machine learning models that enhance early detection and classification of lung cancer. Through meticulous curation and rigorous preprocessing, this dataset showcases the transformative potential of AI in healthcare, underscoring its role in improving diagnostic accuracy and efficiency.

### Image preprocessing

The preprocessing of images stands as a pivotal stage in the pipeline of developing a machine learning model, especially when handling medical imaging data like the IQ-OTHNCCD lung cancer dataset. This procedure comprises several crucial steps, each tailored to convert the raw CT scan images into a format conducive to effective analysis by a convolutional neural network (CNN).

Initially, image resizing is conducted. Given the inherent variability in the dimensions of CT scans, it is imperative to standardize the size of all images to ensure consistent input to the CNN. Resizing is performed while preserving the aspect ratio to avoid distortion, typically scaling down to a fixed size (e.g., 256 × 256 pixels). This uniformity is vital for the neural network to process and glean insights from the data effectively, as it necessitates a consistent input size [21].

**Fig. 4:**
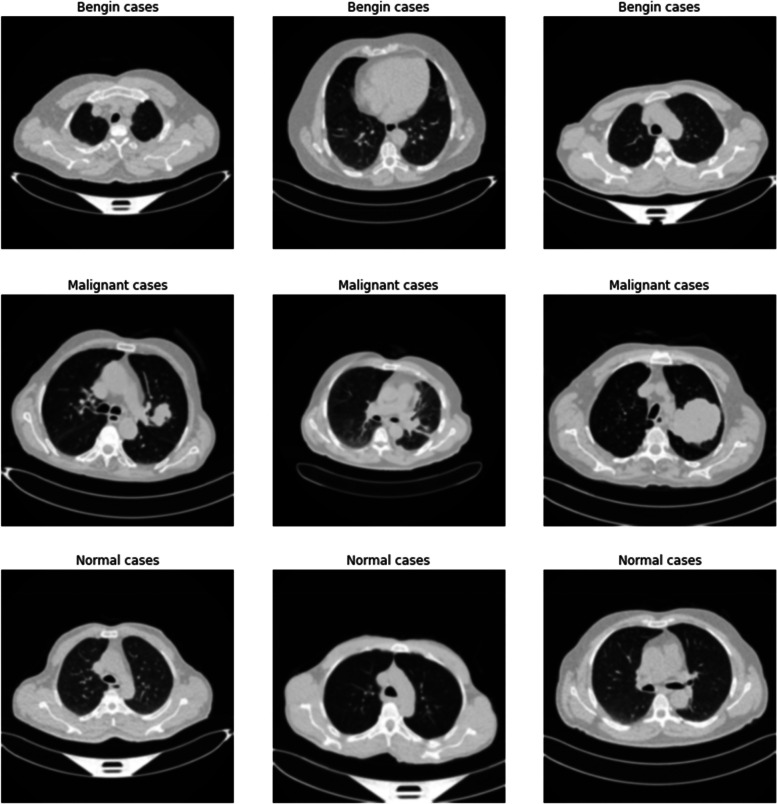
Pre-processed images

Some pre-processed images to enhance the accessibility has been provided in Fig. 4.

Following resizing, normalization of pixel values is performed. CT scans, by nature, contain a wide range of pixel intensities, which can adversely affect the training process of a CNN due to the varying scales of image brightness and contrast. Normalization is a crucial preprocessing step in image analysis that adjusts the pixel values to fall within a specific range, commonly 0 to 1 or -1 to 1. This adjustment is typically achieved by dividing the pixel values by the maximum possible value, which is 255 for 8-bit images. Such a normalization process ensures that the model can train faster and more efficiently. This step ensures that the model trains faster and more effectively, as small, standardized values facilitate quicker convergence during the optimization process.

Gaussian blur is then applied as an additional preprocessing step. This technique, which employs a Gaussian kernel to smooth the image, is instrumental in reducing image noise and mitigating the effects of minor variations and artifacts in the scans. By doing so, the model’s focus is directed toward the salient features relevant to lung cancer classification, rather than being distracted by irrelevant noise or details. Gaussian blur operates by convolving the image with a Gaussian function, effectively averaging the pixel values within a specified radius. This process smoothens the image, reducing high-frequency components and noise, which can otherwise lead to overfitting or distraction during the training of the CNN.

In the context of lung cancer CT scans, Gaussian blur helps to highlight the important structural elements of the lungs and nodules while suppressing irrelevant details that could complicate the model’s learning process. By smoothing the images, Gaussian blur enhances the model’s ability to generalize by focusing on the more significant, lower-frequency features of the image, such as the shape and size of nodules, rather than being confounded by small variations or noise. This is particularly beneficial in medical imaging, where the presence of noise and artifacts can obscure critical diagnostic features.

The application of Gaussian blur can also aid in generalizing the model, preventing overfitting to the high-frequency noise present in the training set. It is achieved using Eq. 3 and the SMOTE ratio through Eq. 4.

3 $$\text{Gaussian Blur}=\text{Image}*\text{Gaussian Kernel}$$

4 $$\text{SMOTE Ratio}=\frac{\text{Number of Synthetic Samples}}{\text{Number of Real Samples}}$$

These are the preprocessing steps collectively enhance the quality and consistency of the input data, enabling the CNN to focus on learning meaningful, discriminative features from the CT images [22]. By ensuring that the images are appropriately resized, normalized, and filtered, the model is better equipped to identify the subtle nuances associated with different stages of lung cancer, thereby improving its diagnostic accuracy and reliability. Through meticulous image preprocessing, the foundation is laid for developing a robust machine learning model capable of contributing significantly to the field of medical imaging and diagnostics.

### Deep learning model

The model architecture utilized in this study is a Convolutional Neural Network (CNN), renowned for its effectiveness in various image analysis tasks, notably in the domain of medical image processing. In this study, we utilized a Convolutional Neural Network (CNN) architecture, known for its effectiveness in analyzing images, particularly in medical contexts like lung cancer diagnosis from CT scans. Let’s break down how it works in simpler terms. First, the input layer takes in images resized to a standard size of 256 × 256 pixels, in black and white. This consistency helps the CNN learn efficiently. Then comes the first convolutional layer, where the model looks for basic patterns like edges and textures using small 3 × 3 filters. After that, a process called max pooling reduces the image’s size, focusing on the most important features. This step helps the model generalize better and ignore noise. We repeat this process with another convolutional layer to capture more complex patterns. The flattened layer turns the extracted features into a format the model can understand. Next, a fully connected layer reasons based on these features, helping with the final classification. The output layer then gives probabilities for each class (benign, malignant, or normal). Throughout training, we used the Adam optimizer to adjust learning rates and manage gradients effectively. Additionally, we applied a technique called SMOTE to balance our dataset, ensuring the model learned from all classes equally. By carefully designing our CNN architecture and incorporating these steps, we aimed to create a model that can accurately classify lung cancer stages from CT scans.

- **Input layer**: The input layer accepts images resized to 256 × 256 pixels, maintaining a single channel (grayscale), resulting in an input shape of (256, 256, 1).
- **First convolutional layer**: This layer consists of 64 filters of size 3 × 3, using a ReLU (Rectified Linear Unit) activation function. The choice of 64 filters is aimed at capturing a broad array of features from the input image, while the 3 × 3 filter size is standard for capturing spatial relationships in the image data. The equation involved are given in Eqs. 5 and 6.
  5 $$Convolution\,operation:({z}_{i,j,k}^{\left[1\right]}=\sum\limits_{l=0}^{2}\sum\limits_{m=0}^{2}\sum\limits_{n=1}^{64}{W}_{l,m,n,k}^{\left[1\right]}\times {a}_{i+l,j+m,n}^{\left[0\right]}+{b}_{k}^{\left[1\right]}$$
  6 $$Activation\,function:({a}_{i,j,k}^{\left[1\right]}=\text{max}\left(0,{z}_{i,j,k}^{\left[1\right]}\right)) \left(ReLU\right)$$
- **First max pooling layer**: Following the convolutional layer, the model incorporates a max pooling layer with a 2 × 2 pool size. This layer serves to decrease the spatial dimensions of the feature maps, which not only helps in reducing the computational load but also enhances the model’s generalization capabilities. By focusing on the most prominent features, max pooling ensures that the model does not overfit to the noise in the training data. It is done using Eq. 7.
  7 $$Pooling\,operation:({a}_{i,j,k}^{\left[1\right]}=\underset{l,m}{\text{max}}{a}_{2i+l,2j+m,k}^{\left[1\right]})$$
- **Second convolutional layer**: Another set of 64 filters is applied, like the first convolutional layer, to further refine the feature extraction. This layer also uses a 3 × 3 kernel and is followed by a ReLU activation. It is achieved using Eqs. 8 and 9.
  8 $$Convolution\,operation:({z}_{i,j,k}^{\left[2\right]}=\sum\limits_{l=0}^{2}\sum\limits_{m=0}^{2}\sum\limits_{n=1}^{64}{W}_{l,m,n,k}^{\left[2\right]}\times {a}_{i+l,j+m,n}^{\left[1\right]}+{b}_{k}^{\left[2\right]})$$
  9 $$Activation\,function:({a}_{i,j,k}^{\left[2\right]}=\text{max}\left(0,{z}_{i,j,k}^{\left[2\right]}\right))\left(ReLU\right)$$
- **Second max pooling layer**: This layer additionally decreases the size of the feature maps, aiding in the prevention of overfitting and lessening the computational burden.
- **Flattening**: The feature maps are flattened into a single vector to prepare for the fully connected layers, facilitating the transition from convolutional layers to dense layers.
- **Fully connected layer**: A dense layer with 16 neurons is used, providing a high-level reasoning based on the extracted features. This layer utilizes a linear activation function to allow for a range of linear responses. The equations helping in this are given in Eqs. 10 and 11.
  10 $$Operation:({z}^{\left[3\right]}={W}^{\left[3\right]}\cdot {a}^{\left[2\right]}+{b}^{\left[3\right]})$$
  11 $$Activation\,function:({a}^{\left[3\right]}={z}^{\left[3\right]})\left(linear\,activation\right)$$
- **Output layer**: The final layer of the model contains three neurons, each representing one of the classes: benign, malignant, and normal. It uses a SoftMax activation function, which is selected because it provides a probability distribution across these three classes, making it . involved are given in Eqs. 12 and 13.
  12 $$Operation:({z}^{\left[4\right]}={W}^{\left[4\right]}\cdot {a}^{\left[3\right]}+{b}^{\left[4\right]})$$
  13 $$Activation\,function:({a}_{i}^{\left[4\right]}=\frac{{e}^{{z}_{i}^{\left[4\right]}}}{{\sum }_{j=1}^{3}{e}^{{z}_{j}^{\left[4\right]}}})\left(softmax\right)$$
- **Optimizer**: The Adam optimizer is used due to its effectiveness in managing sparse gradients and its ability to adapt learning rates, which enhance the convergence speed during training. The equation involved in this is given in Eq. 14.
  14 $$Update \,rule\left(Adam\right):({{\uptheta }}_{t+1}={{\uptheta }}_{t}-\frac{{\upeta }}{\sqrt{\widehat{{v}_{t}}}+\mathrm\epsilon \text{}}\widehat{{m}_{t}})$$
  $$where \left({\uptheta }\right) \, represents \,the \,parameters\left(weights \,and \,biases\right),$$
  $$\left({\upeta }\right) is \,the \,learning \,rate,$$
  $$\left(\widehat{{m}_{t}}\right)is \,the \,first \,moment \,estimate,$$
  $$\left(\widehat{{v}_{t}}\right) \,is \,the \,second \,moment \,estimate,$$
  $$and\;\left(\in\right)\,is\,a\,small\,constant\,to\,prevent\,division\,by\,zero.$$

CNN is chosen for its proven efficacy in image classification tasks, particularly its ability to learn hierarchical patterns in data. In medical imaging, CNNs have demonstrated success in identifying subtle patterns that are indicative of various pathologies, making them ideal for this application. The sequential model with convolutional layers followed by pooling layers allows for the extraction and down sampling of features, which is critical for capturing relevant information from medical images.

The Synthetic Minority Over-Sampling Technique (SMOTE) represents an innovative strategy devised to address the issue of class imbalance within the dataset. Class imbalance poses a substantial risk of biasing the model’s performance, particularly in medical datasets where one class may be underrepresented. SMOTE functions by creating synthetic samples within the feature space of the minority class, drawing inspiration from the feature space of its nearest neighbors. This process aids in rectifying class imbalances and ensuring more equitable representation during model training.

**Fig. 5:**
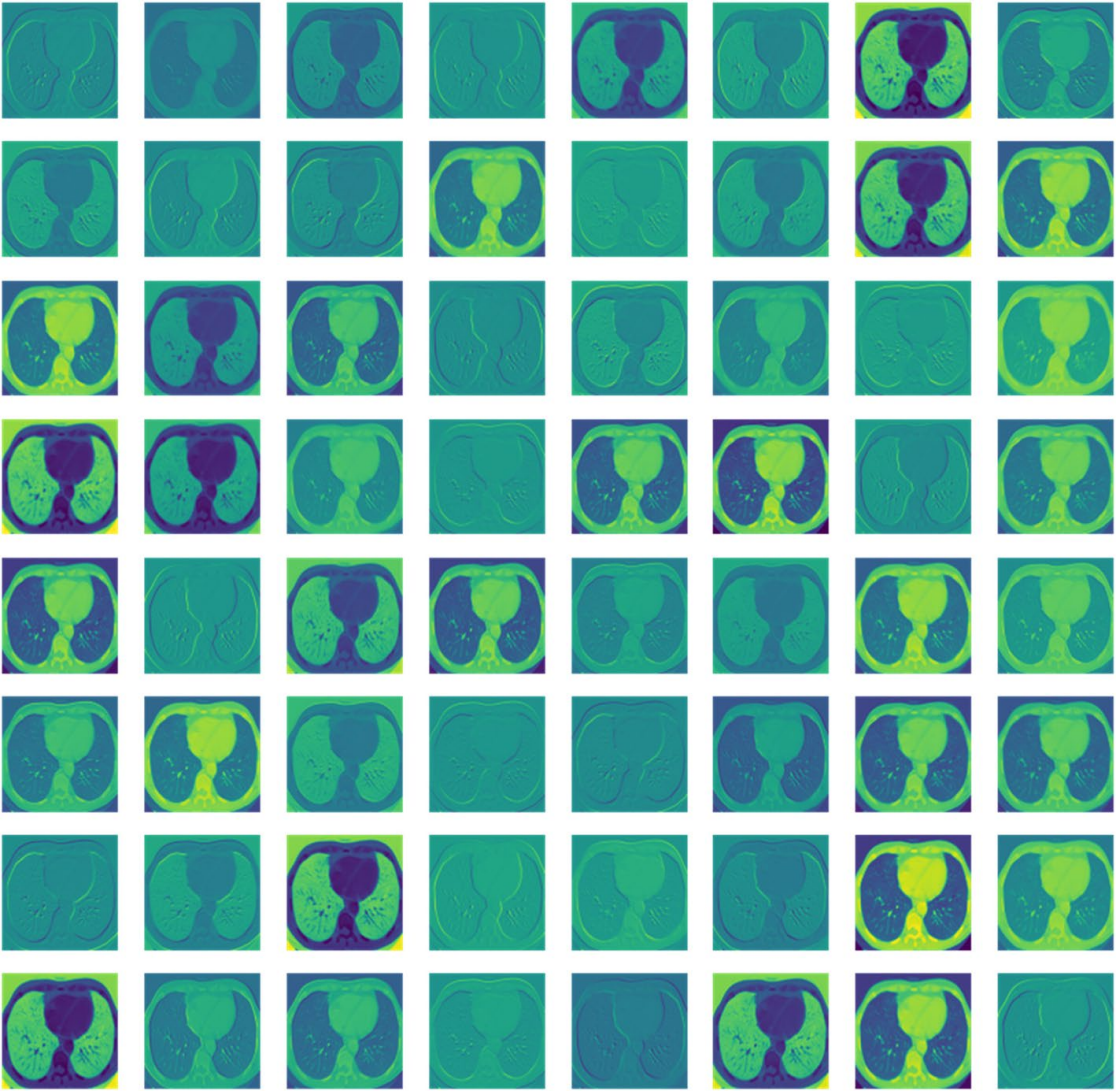
Filter map

Filter mapping of a sample image is shown in Fig. 5 to make it more sound about the interoperability of the model.

In this research:

- **Application of SMOTE**: SMOTE is applied only to the training data to prevent information leakage and to promote robust generalization on unseen data. It balances the dataset by augmenting the minority classes, ensuring that the model does not become biased toward the majority class.
- **Impact on model performance**: By addressing the class imbalance, SMOTE helps in improving the model’s sensitivity towards the minority class, which is crucial in medical diagnostics, as overlooking a positive case can have serious implications.
- **Considerations**: While SMOTE can significantly improve model performance in cases of class imbalance, it’s essential to monitor for overfitting, as the synthetic samples may cause the model to overgeneralize from the minority class.

The algorithm for the proposed model is presented in Algorithm 1.

**Figure:**
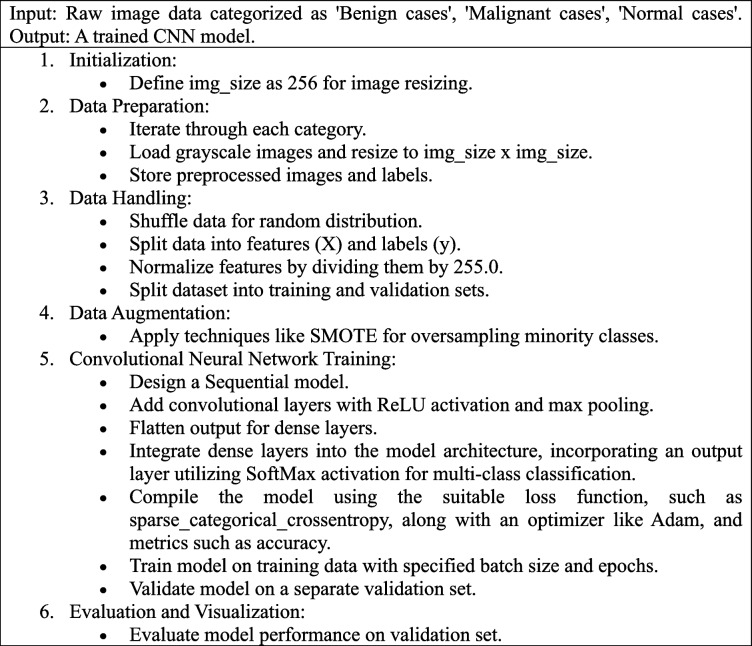
**Algorithm 1:** Proposed algorithm for the methodology

As per the algorithm in the initial convolutional layers of the model, two sets of convolutional layers followed by max-pooling layers play a pivotal role in feature detection. Utilizing a standard 3 × 3 kernel size allows the model to discern small, localized features within CT scan images. By stacking these convolutional layers before applying max pooling, the model effectively captures intricate patterns such as edges, textures, and shapes, crucial for distinguishing between benign, malignant, and normal lung tissue. The ReLU activation function is employed in these convolutional layers due to its effectiveness in introducing non-linearity, enabling the model to learn complex patterns efficiently. Additionally, max pooling is utilized to downsample the feature maps, reducing computational load and enhancing robustness to image variations, thereby improving translational invariance. Following feature extraction, the model flattens the output and transitions to dense layers, condensing learned information into abstract representations. The final layer consists of three neurons, representing the three classes under consideration, employing the SoftMax activation function to transform logits into probabilities, thereby providing insights into the model’s confidence regarding each class. Throughout the compilation and training phases, the Adam optimizer and sparse categorical crossentropy loss function, as depicted by Eq. 15, are chosen due to their adaptive learning rate features and appropriateness for classification objectives. Validation on an independent dataset is crucial for detecting overfitting and refining hyperparameters.

15 $$\text{Cross entropy Loss}=-\sum\limits_{i}^{n}\left({y}_{i}\text{log}\left({p}_{i}\right)+\left(1-{y}_{i}\right)\text{log}\left(1-{p}_{i}\right)\right)$$

In the training phase, SMOTE is strategically applied to create a balanced dataset representative of all classes, crucial for generalizing well across various lung tissue conditions, especially in medical datasets where class imbalance may exist.

### Training and validation

Throughout the training and validation phases of the deep learning model, meticulous steps are taken to ensure that the model not only learns effectively from the training data but also demonstrates robust generalization capabilities when presented with new, unseen data. This phase plays a pivotal role in evaluating the model’s proficiency in accurately classifying lung cancer stages from CT scans.

The training process initiates with the segmentation of the dataset into distinct training and validation subsets. This segmentation is performed in a stratified manner to guarantee that each subset encompasses a balanced representation of the various classes. Such stratification is essential for maintaining consistency and mitigating biases, particularly in light of the class imbalance addressed by SMOTE during training. Approximately 80% of the data is allocated for training purposes, while the remaining 20% is reserved for validation.

Subsequent to the data segmentation, the training commences with the utilization of a batch size of 8. The selection of a smaller batch size is deliberate, aiming to facilitate more precise and nuanced updates to the model’s weights during each iteration, thereby potentially enhancing generalization. Nonetheless, it is imperative to strike a balance between this granularity and computational efficiency, as smaller batch sizes may prolong the training duration.

**Fig. 6:**
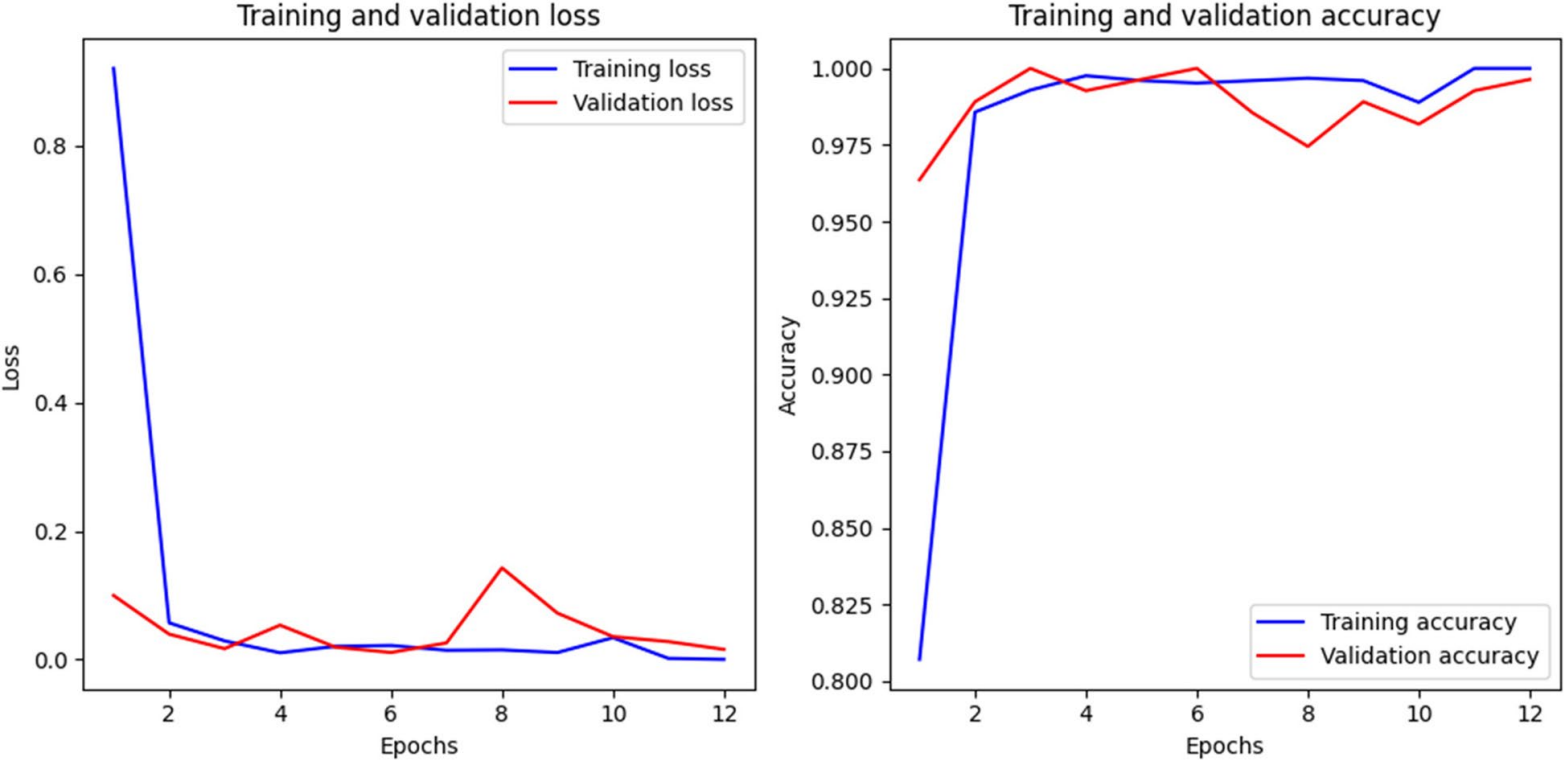
Training and validation loss and accuracy

The number of epochs is predetermined to be 12, indicating the total number of complete passes that the learning algorithm will undertake across the entire training dataset. This choice represents a delicate balance between underfitting and overfitting; insufficient epochs may hinder the model’s learning process, whereas excessive epochs may result in the model memorizing the training data, consequently impairing its ability to generalize effectively. The progression of training and validation loss and accuracy across epochs is visualized in Fig. 6.

During training, the model’s performance is continuously evaluated using a comprehensive set of performance metrics assessed against the validation set. These metrics encompass accuracy, precision, recall, and F1-score, all of which are instrumental in comprehending the model’s strengths and weaknesses in classifying each lung cancer stage. Accuracy furnishes a broad overview of the model’s overall performance, while precision and recall delve deeper into its class-specific performance, a critical consideration in medical diagnostics where false negatives and false positives carry significant consequences. The F1-score serves to harmonize precision and recall, furnishing a unified metric to gauge the model’s equilibrium between these two facets.

Moreover, the validation process incorporates a confusion matrix and ROC curves to furnish a more granular analysis of the model’s performance across diverse thresholds and classes. The confusion matrix delineates the model’s true positives, false positives, false negatives, and true negatives, offering a snapshot of its classification capabilities. Meanwhile, ROC curves and the corresponding AUC (Area Under the Curve) provide insights into the model’s capacity to discriminate between classes at varying threshold settings, a crucial consideration for refining the model’s decision boundary.

In our quest to maximize the performance of our Convolutional Neural Network (CNN) model for lung cancer classification, we meticulously fine-tuned several critical hyperparameters that play pivotal roles in shaping the learning process and ultimately, the model’s accuracy. Specifically, we focused on optimizing the learning rate, batch size, number of filters in each convolutional layer, filter size, and dropout rate. Firstly, we delved into exploring a spectrum of learning rates to pinpoint the optimal value that ensures swift convergence towards the minimum of the loss function without overshooting. Next, we scrutinized various batch sizes to strike a delicate balance between training time and the stability of the gradient descent process. Moving forward, we embarked on an exploration of different combinations of the number of filters and filter sizes in the convolutional layers, aiming to unearth the configuration most adept at extracting salient features from the intricate CT scan images. Additionally, to combat overfitting and foster model robustness, we meticulously optimized the dropout rate, discerning the precise proportion of neurons to deactivate during training. Our methodology embraced a meticulous grid search strategy, systematically traversing through predefined sets of values for each hyperparameter while evaluating the model’s performance using cross-validation. This exhaustive search enabled us to pinpoint the hyperparameter combination that not only elevated the model’s classification accuracy but also bolstered its generalization capabilities. Subsequently, the efficacy of the selected hyperparameters was meticulously validated using a distinct validation set, underscoring the robustness and reliability of our chosen parameters. Through this systematic and rigorous approach to hyperparameter tuning, we achieved remarkable strides in fortifying the performance and stability of our lung cancer classification model, thereby augmenting its potential for real-world clinical applications.

The training and validation phases operate iteratively, with refinements made to the model’s architecture, hyperparameters, or training methodology based on the validation outcomes. This iterative refinement persists until the model achieves a satisfactory equilibrium of accuracy, generalizability, and robustness, thereby ensuring its efficacy and reliability in clinical settings for lung cancer stage classification.

### Statistical methods

In the analysis of the IQ-OTH/NCCD lung cancer dataset, various statistical and machine learning techniques were employed to ensure a comprehensive evaluation of the data. The primary focus was on classification metrics to assess the performance of the predictive models.

- **Confusion matrix**: The confusion matrix serves as a pivotal component in our analysis, furnishing a visual representation of the model’s performance. It succinctly presents the counts of true positives, true negatives, false positives, and false negatives, thereby offering a lucid comprehension of the model’s classification accuracy and any instances of misclassification.
- **Accuracy**: The accuracy metric was calculated by dividing the number of correctly predicted observations by the total number of observations, providing a straightforward measure for assessing the model’s overall performance. However, relying solely on accuracy can be deceptive, particularly in datasets with imbalanced class distributions. Therefore, it is imperative to incorporate additional metrics for a more comprehensive evaluation. It is achieved by Eq. 16.
  16 $$\text{Accuracy}=\frac{\text{Number of Correct Predictions}}{\text{Total Number of Predictions}}\times 100\%$$

- **Precision (positive predictive value)**: Precision was utilized to assess the accuracy of positive predictions, quantified as the ratio of true positives to the sum of true positives and false positives. This metric bears significant relevance in scenarios where the repercussions of false positives are considerable. It is achieved by Eq. 17.
  17 $$\text{Precision}=\frac{\text{True Positives}}{\text{True Positives}+\text{False Positives}}$$

- **Recall (sensitivity or true positive rate)**: Recall assesses the model’s ability to detect positive instances, calculated as the ratio of true positives to the sum of true positives and false negatives. This metric holds particular importance in medical diagnostics, where failing to identify a positive case can lead to severe consequences. It is achieved by Eq. 18.
  18 $$\text{Recall}=\frac{\text{True Positives}}{\text{True Positives}+\text{False Negatives}}$$

- **F1-score**: The F1-score, which is the harmonic mean of precision and recall, was used to provide a balance between the two metrics, particularly valuable in situations of class imbalance. It is a more robust measure than accuracy in scenarios where false negatives and false positives have different implications. It is achieved by Eq. 19.
  19 $$\text{F1-Score}=2\times \frac{\text{Precision}\times \text{Recall}}{\text{Precision}+\text{Recall}}$$

- **Cohen’s kappa**: The Cohen’s Kappa statistic was applied to assess the agreement between observed and predicted classifications, accounting for chance agreement. This statistic offers a nuanced understanding of the model’s performance, which is particularly valuable in scenarios involving imbalanced datasets. It is achieved by Eq. 20.
  20 $$\text{Cohen's Kappa}=\frac{{p}_{o}-{p}_{e}}{1-{p}_{e}}$$

- **Mean Squared Error (MSE) and Root Mean Squared Error (RMSE)**: MSE (Mean Squared Error) and RMSE (Root Mean Squared Error) were calculated to evaluate the average squared difference and the square root of the average squared differences, respectively, between predicted and actual classification categories. These metrics are instrumental in understanding the variance of prediction errors. MSE and RMSE are achieved using Eqs. 21 and 22, respectively.
  21 $$\text{MSE}=\frac{1}{n}{\sum }_{i=1}^{n}{\left({y}_{i}-\widehat{{y}_{i}}\right)}^{2}$$
  22 $$\text{RMSE}=\sqrt{\text{MSE}}\left(22\right)$$

- **Mean Absolute Error (MAE)**: MAE (Mean Absolute Error) measures the average magnitude of errors in a set of predictions, regardless of their direction. It is a linear score, meaning that all individual differences are equally weighted in the average. It is achieved using Eq. 23.
  23 $$\text{MAE}=\frac{1}{n}{\sum }_{i=1}^{n}\left|{y}_{i}-\widehat{{y}_{i}}\right|$$

- **Receiver Operating Characteristic (ROC) Curve and Area Under the Curve (AUC)**: The ROC curve graphically illustrates the diagnostic ability of the model by plotting the true positive rate against the false positive rate at various threshold settings. The AUC (Area Under the Curve) provides a single scalar value summarizing the overall performance of the model across all possible classification thresholds. It is achieved using Eq. 24.
  24 $$\text{AUC}={\int }_{0}^{1}\text{ROC Curve}\left(t\right)dt$$

- **F2-score**: The F2-score was calculated to weigh recall higher than precision, useful in scenarios where missing positive predictions is more detrimental than making false positives. It is achieved using Eq. 25.
  25 $$\text{F2-Score}=\left(1+{2}^{2}\right)\times \frac{\text{Precision}\times \text{Recall}}{\left({2}^{2}\times \text{Precision}\right)+\text{Recall}}$$

These statistical methods and metrics provided a multifaceted evaluation of the model’s performance, ensuring a robust analysis of the predictive capabilities and reliability in classifying the cases within the IQ-OTH/NCCD lung cancer dataset.

## Results

**Fig. 7:**
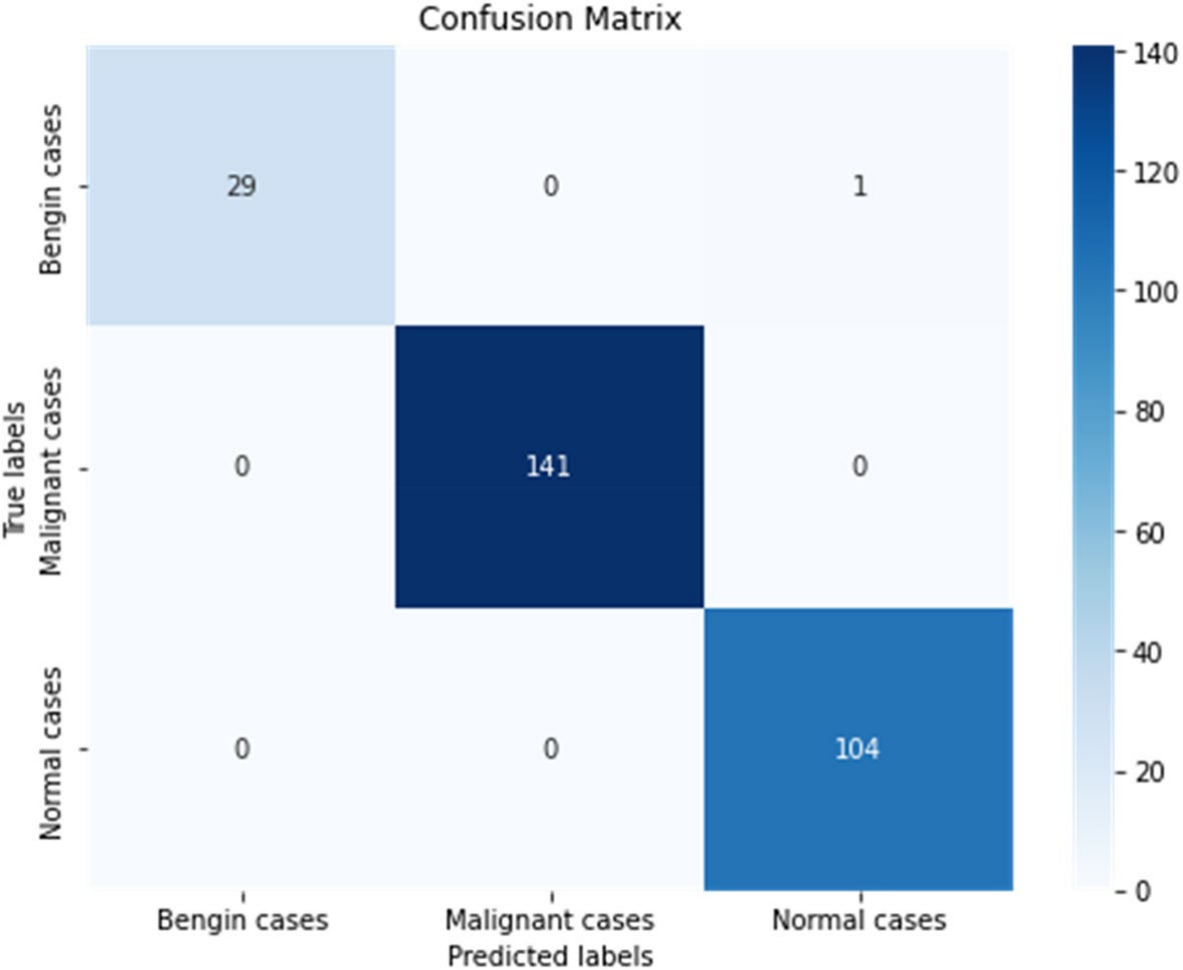
Confusion matrix

**Fig. 8:**
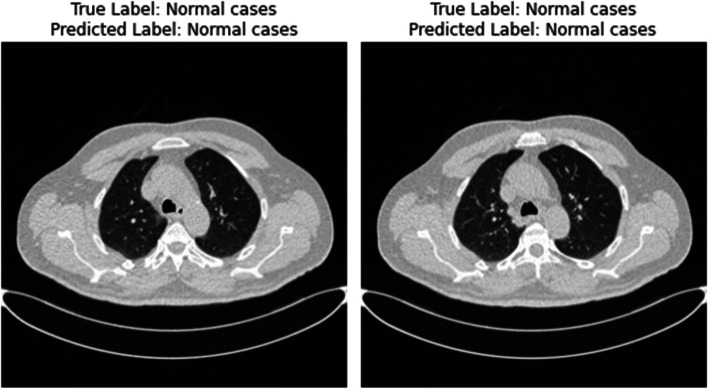
Correctly classified instances

The evaluation of the IQ-OTH/NCCD lung cancer dataset through our predictive model yielded detailed insights across various statistical metrics, showcasing the model’s efficacy in classifying lung cancer stages. Here we delve into a comprehensive analysis of each metric:

- **Confusion matrix**: The confusion matrix offered a detailed perspective on the model’s classification performance, unveiling a notable count of true positives and true negatives, reflecting precise predictions. Notably, there were minimal occurrences of false positives and false negatives, underscoring the model’s accuracy in discerning between benign, malignant, and normal cases. The same is visualized in Fig. 7.

- **Accuracy**: The overall model accuracy was noted at 99.64%, highlighting the model’s robust capacity to accurately identify and classify instances within the dataset. This exceptional accuracy rate underscores the model’s reliability in clinical diagnostic settings, establishing a solid basis for subsequent validation and potential clinical implementation. To provide visual insight of this Fig. 8 gives truly classified instances.

- **Precision**: The precision metric provided valuable insights into the model’s predictive reliability. It attained a precision of 96.77% for benign cases, signifying a high probability that a case predicted as benign is indeed benign. Moreover, for malignant and normal cases, the precision reached 100%, demonstrating the model’s outstanding ability to predict these categories accurately without any false positives.
- **Recall**: The recall scores were equally remarkable, achieving 100% for both benign and malignant cases, and 99.04% for normal cases. These findings underscore the model’s sensitivity and its capability to accurately detect all true positive cases, thereby mitigating the risk of false negatives as a pivotal consideration in medical diagnostics.
- **F1-score**: The F1-scores, which strike a balance between precision and recall, were 98.36% for benign, 100% for malignant, and 99.52% for normal cases. These scores signify the model’s balanced performance, guaranteeing both the accuracy of positive predictions and the reduction of false negatives. To enhance the visualization of the classification report, Table 3 provides a statistical representation.

**Table 3:** Classification report

	Precision	Recall	F1-score
**Malignant**	0.9677	1	0.9836
**Benign**	1	1	1
**Normal**	1	0.9904	0.9952

**Fig. 9:**
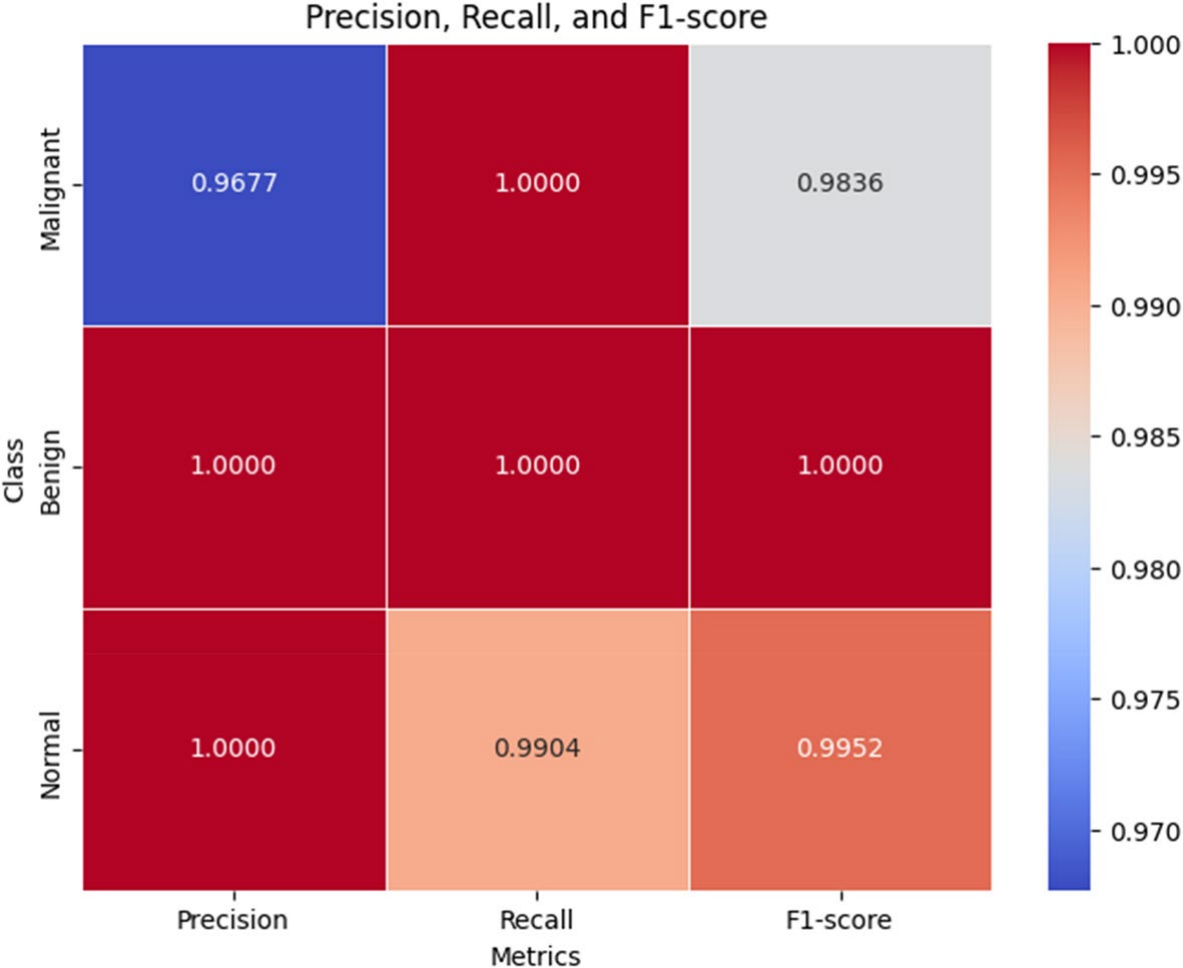
Classification report

Based on Table 3 a heatmap to visualize the same detail is provided in Fig. 9 for better insights.

**Fig. 10:**
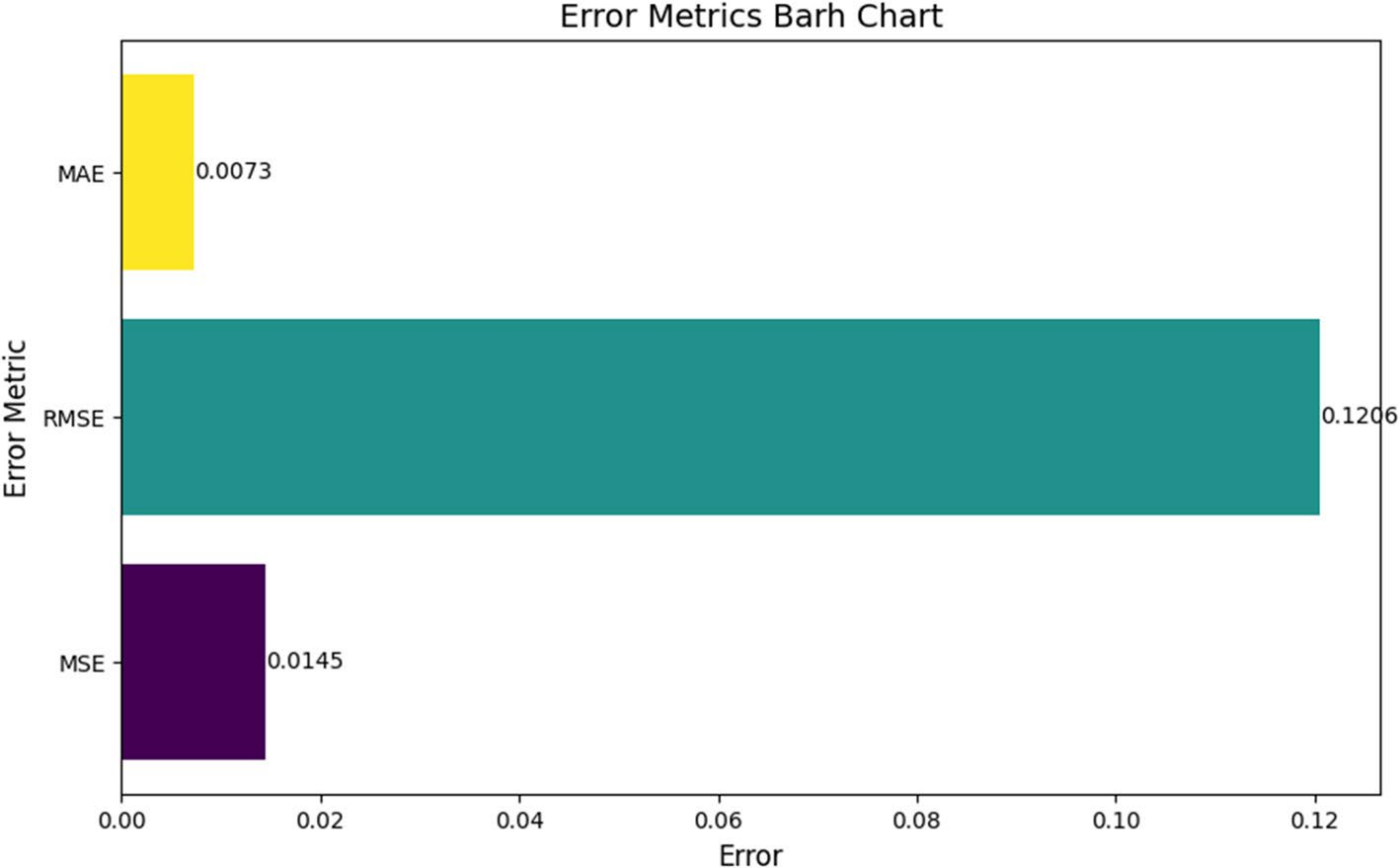
Error metrics barh chart

**Fig. 11:**
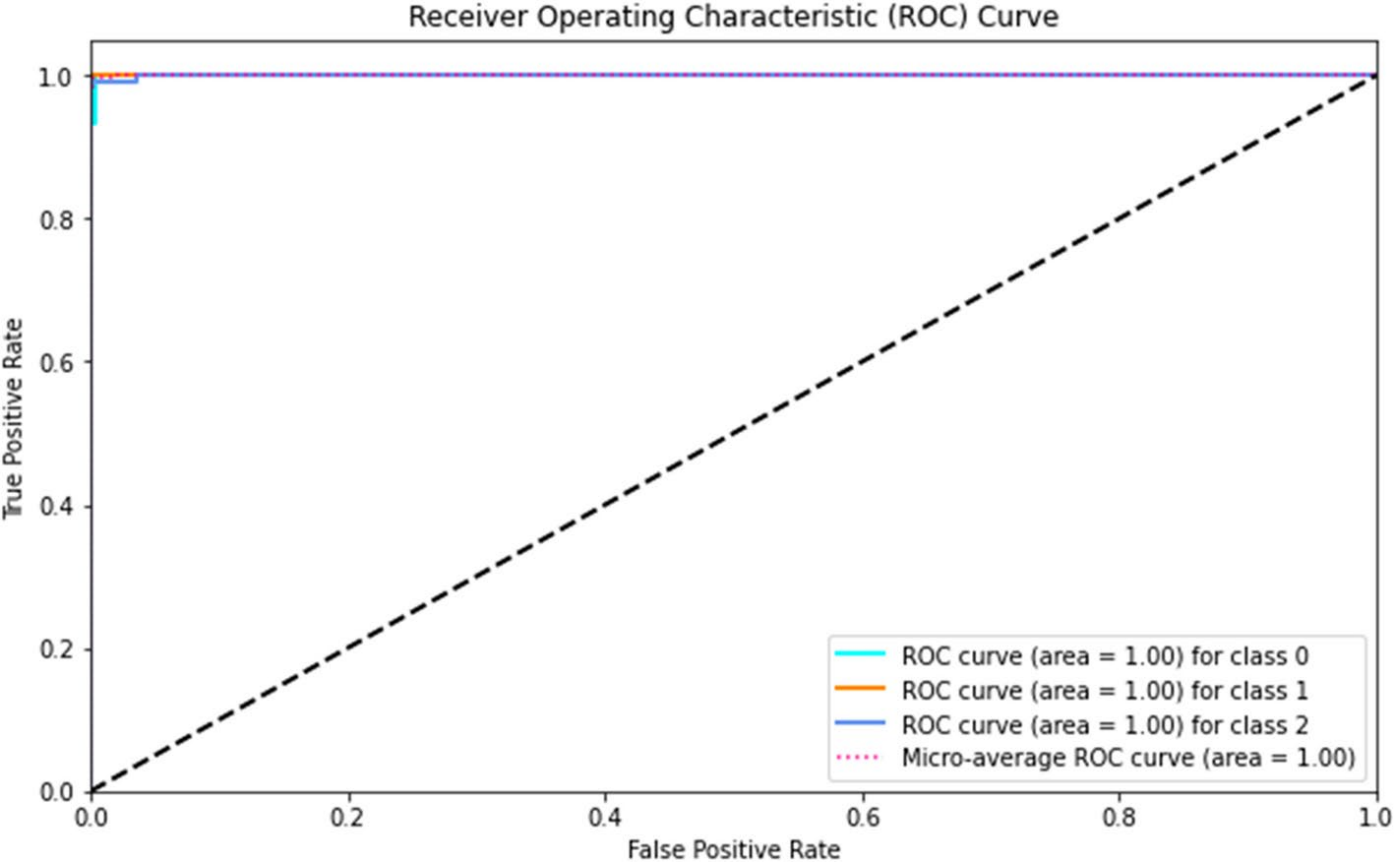
ROC curve

**Fig. 12:**
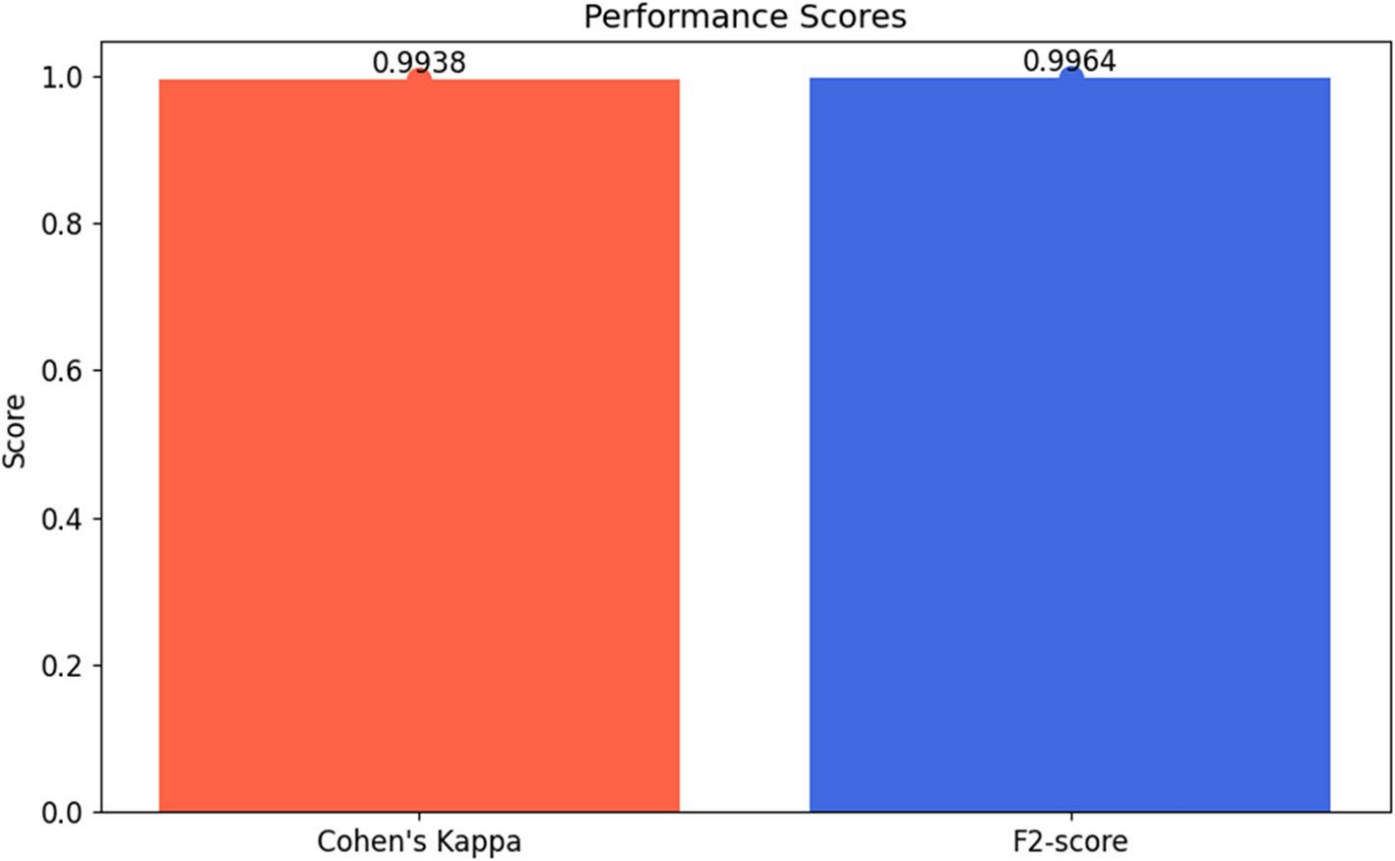
Performance scores

- **Cohen’s kappa**: With a Cohen’s Kappa score of 0.9938, the model exhibited perfect agreement with the actual classifications, surpassing the performance expected by chance alone. This underscores an elevated level of consistency in the model’s predictions, thus reinforcing its reliability.
- **Mean Squared Error (MSE) and Root Mean Squared Error (RMSE)**: The model reported an MSE of 0.0145 and an RMSE of 0.1206, indicating minimal variance and bias in the prediction errors. These low values suggest that the model’s predictions are consistently close to the actual values, enhancing trust in its predictive power.
- **Mean Absolute Error (MAE)**: With an MAE of 0.0073, the model exhibited minimal average error magnitude in its predictions, signifying high predictive accuracy. This metric further reinforces the model’s suitability for clinical settings where precision is crucial. To visualize the error metrics, a bar chart is given in Fig. 10.

- **Receiver Operating Characteristic (ROC) Curve and Area Under the Curve (AUC)**: The ROC curves and corresponding AUC values were exceptional, achieving AUCs of 1.00 for malignant, benign, and normal cases. These results indicate the model’s outstanding discrimination ability between different classes across various threshold settings. The roc-auc curve is provided in Fig. 11.

- **F2-score**: The F2-score of 0.9964, which places more emphasis on recall, indicates the model’s strong ability to identify positive cases. This is particularly important in the medical field, where failing to detect a condition could have profound consequences. The visual representation of performance score is given in Fig. 12.

The detailed results across these metrics provide a comprehensive picture of the model’s performance, highlighting its precision, reliability, and robustness in classifying lung cancer stages from the IQ-OTH/NCCD dataset. The findings demonstrate the model’s potential as a diagnostic tool, supporting its further investigation and potential integration into clinical practice.

## Discussion

The analysis of the IQ-OTH/NCCD lung cancer dataset with our model reveals a groundbreaking level of performance in medical image classification. With an accuracy of 99.64% and exceptional precision and recall metrics across the three categories (benign, malignant, and normal), the model emerges as a highly reliable diagnostic aid. The significance of these results extends beyond the high metric scores; it lies in the model’s capability to accurately distinguish between benign and malignant cases, a critical aspect for patient management and treatment planning.

The high F1-score underscores the model’s balanced consideration of precision and recall, thereby minimizing the risk of misdiagnosis. Additionally, the emphasis on recall in the F2-score holds particular significance in the medical domain, where overlooking a positive case (false negative) can have more severe consequences than erroneously identifying a case as positive (false positive). The comparison between the baseline models and proposed model has been given in Table 4.

**Table 4:** Comparison with existing studies

Study	Techniques	Accuracy
**Asghar Ali Shah et al. (2023)** [23]	Convolutional Neural Networks (CNNs) and Ensemble	95%
**Mohammad A. Alzubaidi et al. (2021)** [24]	SVM with HOG features	88%
**Dimitrios Mathios et al. (2021)** [25]	Cell-free DNA fragmentomes	94%
**Shahid Mehmood et al. (2022)** [26]	Transfer learning	98.4%
**Elias Dritsas, Maria Trigka (2022)** [27]	Rotation Forest model	97.1%
**Mehedi Masud et al. (2021)** [28]	Deep learning and digital image processing	96.33%
**Iftkhar Naseer et al. (2023)** [29]	Modified U-Net Based Lobe Segmentation and detection	97.70%
**Bharathy S, Pavithra R, Akshaya. B (2022)** [30]	Random Forest algorithm	88.5%
**Gopi Kasinathan and Selvakumar Jayakumar (2022)** [31]	Hybrid technique for PET/CT images	98.6%
**Das, S., et al. (2023)** [32]	CNN and Inception V3	93.44%
**Tasnim, Nowshin, et al. (2024)** [33]	CNN, Resnet50, and InceptionV3	98%
**Safta, Wiem, and Ahmed Shaffie (2024)** [34]	Integration of 3D-Local Octal Pattern (LOP) descriptor, 3D-Convolutional Neural Network (CNN), and geometric feature analysis	97.84%
**Khaliq, Kiran, et al. (2023)** [35]	Transfer learning with Densely Connected Convolutional Networks (DenseNet-121)	99%
**Nigudgi, Surekha, and Channappa Bhyri. (2023)** [36]	Transfer learning with hybrid model (AlexNet, VGG, GoogleNet)	97%
**Proposed Model**	Double Layered CNN with Advanced Image Processing	99.64%

In the realm of lung cancer detection, many existing models focus predominantly on binary classification, often neglecting the nuanced differentiation between benign and malignant cases [37]. Our model’s tri-classification capability sets a new benchmark, offering a more detailed diagnostic tool compared to the binary classifiers. When juxtaposed with existing methods, our model’s performance underscores its advanced detection capabilities, potentially offering a more nuanced and informative diagnostic perspective than currently available tools.

For clinical practice, the integration of such a high-performing model could revolutionize lung cancer diagnostics [22, 38]. It can augment radiologists’ capabilities, reducing diagnostic time and increasing throughput. The ability to accurately classify lung nodules as benign, malignant, or normal could significantly reduce unnecessary interventions, minimizing patient exposure to invasive procedures and associated risks. Additionally, it can streamline the patient pathway, ensuring rapid treatment initiation for malignant cases and appropriate follow-up for benign conditions [39, 40].

**Fig. 13:**
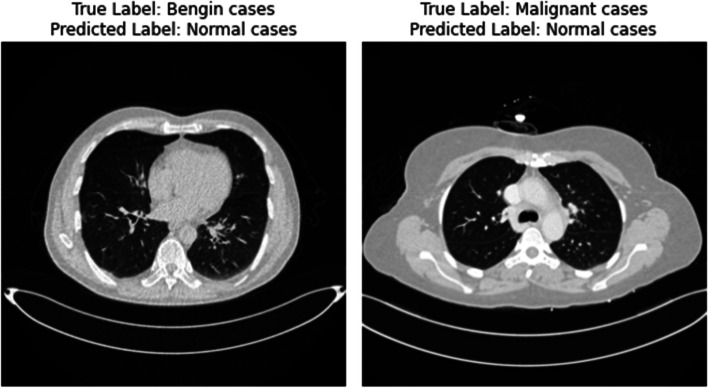
Misclassified instances

While the results are promising, the study’s limitations warrant consideration. The model’s training on a dataset from a specific demographic and geographic area raises questions about its applicability to broader populations. Additionally, the model’s performance in a controlled study environment might not fully translate to the diverse and unpredictable nature of clinical settings. The black-box nature of deep learning models also poses a challenge in clinical contexts, where understanding the rationale behind a diagnosis is as crucial as the diagnosis itself [41]. To make it more clear in Fig. 13 some misclassified instances has been shown.

When evaluating our CNN model’s performance on the lung cancer dataset, we noticed some errors in classification. These mistakes can happen for various reasons. Firstly, some features in the CT scans may look similar between benign and malignant nodules, making it hard for the model to tell them apart. Also, noise and artifacts in the scans can confuse the model by hiding important details. Even though we tried to balance the classes, rare cases could still be challenging for the model to recognize. Plus, early-stage cancer might look very similar to normal tissue, making it tricky for the model to spot. Differences in how scans are taken can also affect the model’s understanding, leading to errors. Lastly, if the model learns too much from the training data, it might not perform well on new, unseen images. To fix these issues, we’re planning to use better techniques for preparing the data, like removing noise more effectively and making the model more flexible to different imaging conditions. We also aim to combine multiple models and use more diverse data to improve accuracy. By addressing these challenges, we hope to make our model better at classifying lung cancer stages.

While the IQ-OTHNCCD lung cancer dataset has been instrumental in developing and validating our model, it is important to recognize its limitations, particularly concerning demographic and geographic diversity. The dataset predominantly represents a specific population, which may not capture the full spectrum of variations seen in global populations. This limitation poses challenges for the model’s generalizability, as differences in demographics, such as age, ethnicity, and underlying health conditions, can influence the presentation of lung cancer in CT scans.

To address these limitations, future research should focus on expanding the dataset to include a more diverse range of CT scan images from various demographic groups and geographic regions. This expansion can be facilitated through collaborations with international medical institutions and accessing publicly available medical imaging repositories. Additionally, incorporating advanced data augmentation techniques that simulate variations in demographic characteristics, such as age and gender, can further enhance the dataset’s diversity. By broadening the dataset, we aim to improve the model’s robustness and ensure its applicability across different populations, ultimately enhancing the utility and reliability of our diagnostic tool in diverse clinical settings. This approach will contribute to developing a more inclusive and universally applicable model for lung cancer diagnosis.

###  Sensitivity analysis of precision, recall, and F1-score

In our endeavor to comprehensively assess the performance of our Convolutional Neural Network (CNN) model for lung cancer diagnosis, we conducted a sensitivity analysis focusing on precision, recall, and the F1-score. Precision sensitivity involved systematically adjusting the threshold values used for classification to observe its impact on false positive rates and the model’s conservatism in identifying positive cases. As precision increased, indicating a more stringent classification approach, false positives decreased, but the risk of false negatives rose, necessitating a delicate balance in medical diagnostics. Conversely, recall sensitivity entailed modifying the model’s sensitivity to detect positive cases, thereby influencing its ability to minimize false negatives. Heightened recall improved the identification of true positives, crucial for early diagnosis and treatment, albeit with potential increases in false positives, mandating cautious management. Additionally, analyzing the F1-score, a harmonic mean of precision and recall, elucidated its role in balancing false positives and false negatives. Optimizing for a high F1-score underscored a balanced approach, ensuring robust performance across both precision and recall metrics. Overall, the sensitivity analysis underscored the significance of striking a delicate balance between precision, recall, and the F1-score to optimize the model’s performance in clinical settings. By navigating and managing these trade-offs effectively, we can bolster the reliability and efficacy of our model in diagnosing lung cancer, thereby contributing to improved patient outcomes.

### Regulatory considerations for clinical application

Implementing machine learning models in clinical settings involves navigating a complex landscape of regulatory requirements to ensure patient safety, data security, and efficacy. One of the primary regulatory hurdles is obtaining approval from medical device regulatory bodies such as the U.S. Food and Drug Administration (FDA), the European Medicines Agency (EMA), or other relevant national authorities. These regulatory agencies require extensive validation studies to demonstrate the model’s accuracy, reliability, and safety in diagnosing lung cancer. This involves rigorous testing on diverse datasets to ensure the model’s generalizability and performance across different patient populations and clinical scenarios.

Additionally, regulatory guidelines mandate that machine learning models used in healthcare must provide a level of interpretability and transparency. Clinicians need to understand the decision-making process of the model to trust and effectively integrate it into clinical workflows. This requirement for explainability poses a challenge for deep learning models, which are often considered “black boxes.” Therefore, developing methods to elucidate the model’s reasoning, such as feature importance analysis or visual explanations, is crucial for meeting regulatory standards.

Data privacy and security are also significant regulatory concerns, particularly with the implementation of regulations like the General Data Protection Regulation (GDPR) in Europe and the Health Insurance Portability and Accountability Act (HIPAA) in the United States. Ensuring that patient data is anonymized, securely stored, and used ethically is essential for compliance. This includes implementing robust data encryption, access controls, and audit trails to protect sensitive health information from unauthorized access and breaches.

Moreover, post-market surveillance is a critical component of regulatory compliance, requiring continuous monitoring of the model’s performance in real-world clinical settings. This involves tracking the model’s diagnostic accuracy, identifying potential biases, and updating the model as needed to maintain its efficacy and safety over time. Establishing a framework for ongoing evaluation and improvement is essential to meet regulatory requirements and ensure the model’s long-term success in clinical applications.

Addressing these regulatory hurdles necessitates close collaboration between developers, healthcare providers, and regulatory bodies to ensure that machine learning models are safe, effective, and aligned with clinical needs. By adhering to these regulatory frameworks, we can facilitate the successful integration of advanced diagnostic tools into healthcare, ultimately enhancing patient outcomes and advancing the field of medical diagnostics.

Future research directions should focus on external validation of the model across various populations and healthcare settings to ascertain its universality and robustness. Integrating multimodal data, encompassing patient history, genetic information, and other diagnostic results, could enhance the model’s diagnostic precision. Addressing the interpretability of deep learning models could foster greater trust and integration into clinical decision-making processes. Additionally, prospective studies assessing the model’s impact on clinical outcomes, patient satisfaction, and healthcare efficiency would provide invaluable insights into its practical benefits and potential areas for improvement.

## Conclusion

This study presented a comprehensive analysis of the IQ-OTH/NCCD lung cancer dataset using a sophisticated machine learning model, which demonstrated exceptional performance in classifying lung cancer stages. Key findings include a near-perfect accuracy rate of 99.64%, alongside impressive precision and recall metrics across benign, malignant, and normal case classifications. The model’s balanced F1-score and the emphasis on recall in the F2-score further highlight its diagnostic precision and sensitivity. These results signify a substantial advancement in the model’s ability to differentiate between nuanced lung cancer stages, providing a critical tool for early and accurate diagnosis.

The implications of these discoveries on the field of lung cancer diagnostics are profound. The model’s precision in classifying lung cancer stages holds the promise of substantially enhancing diagnostic protocols, thereby refining the accuracy and efficiency of lung cancer detection. This advancement has the potential to facilitate earlier treatment interventions, potentially enhancing patient outcomes and survival rates. Moreover, the model’s capability to differentiate between benign and malignant nodules could mitigate the need for unnecessary invasive procedures, consequently reducing patient risk and healthcare expenditures.

Future research should focus on external validation of the model to ensure its effectiveness across diverse populations and clinical settings. The exploration of model interpretability is crucial for clinical adoption, where understanding the basis for diagnostic decisions is essential. Additionally, integrating the model with other diagnostic data and clinical workflows could enhance its utility and impact.

Prospective studies are needed to evaluate the model’s real-world clinical impact, particularly its ability to improve patient outcomes, streamline diagnostic pathways, and reduce healthcare costs. The potential for the model to be adapted or extended to other types of cancers or medical imaging modalities also represents an exciting avenue for future research.

This study highlights the potential of advanced machine learning models to transform lung cancer diagnostics, providing a more precise, effective, and nuanced approach to detecting and classifying lung cancer. The ongoing advancement and incorporation of such models into clinical settings hold the promise of catalyzing substantial progress in patient care and outcomes within the field of oncology.

## Data Availability

Data used for the findings are publicly available at https://www.kaggle.com/datasets/hamdallak/the-iqothnccd-lung-cancer-dataset.

## References

[1] Nooreldeen R (2021). Current and future development in lung cancer diagnosis. Int J Mol Sci.

[2] Rea G (2023). Beyond visual interpretation: quantitative analysis and artificial intelligence in interstitial lung disease diagnosis expanding horizons in radiology. Diagnostics.

[3] Rajasekar V (2023). Lung cancer disease prediction with CT scan and histopathological images feature analysis using deep learning techniques. Results Eng.

[4] Lanjewar MG, Kamini G, Panchbhai, Panem Charanarur (2023). Lung cancer detection from CT scans using modified DenseNet with feature selection methods and ML classifiers. Expert Syst Appl.

[5] Raza R (2023). Lung-EffNet: lung cancer classification using EfficientNet from CT-scan images. Eng Appl Artif Intell.

[6] Chaunzwa TL (2021). Deep learning classification of lung cancer histology using CT images. Sci Rep.

[7] Chaturvedi P, Jhamb A, Vanani M, Nemade V. Prediction and Classification of Lung Cancer Using Machine Learning Techniques. IOP Conference Series: Materials Science and Engineering. 2021;1099:012059. 10.1088/1757-899X/1099/1/012059.

[8] Hong M (2021). Multi-class classification of lung diseases using CNN models. Appl Sci.

[9] Phankokkruad M (2021). Ensemble transfer learning for lung cancer detection. 2021 4th international conference on data science and information technology.

[10] Ren Z, Zhang Y, Wang S (2022). LCDAE: data augmented ensemble framework for lung cancer classification. Technology Cancer Research Treatment.

[11] Protonotarios NE (2022). A few-shot U-Net deep learning model for lung cancer lesion segmentation via PET/CT imaging. Biomedical Physics Engineering Express.

[12] Heuvelmans MA, van Ooijen PM, Ather S, Silva CF, Han D, Heussel CP, Oudkerk M (2021). Lung cancer prediction by Deep Learning to identify benign lung nodules. Lung Cancer..

[13] Le NQK, Kha QH, Nguyen VH, Chen YC, Cheng SJ, Chen CY (2021). Machine learning-based radiomics signatures for EGFR and KRAS mutations prediction in non-small-cell lung cancer. Int J Mol Sci.

[14] Xie Y, Meng WY, Li RZ, Wang YW, Qian X, Chan C, Leung ELH (2021). Early lung cancer diagnostic biomarker discovery by machine learning methods. Transl Oncol.

[15] Li Z (2021). Deep Learning Methods for Lung Cancer Segmentation in Whole-Slide Histopathology Images—The ACDC@LungHP Challenge 2019. IEEE J Biomed Health Inform.

[16] Narvekar S, Shirodkar M, Raut T, Vainganka P, Chaman Kumar KM, Aswale S (2022). A Survey on Detection of Lung Cancer Using Different Image Processing Techniques.

[17] Aharonu M, Kumar RL (2022). Convolutional Neural Network based Framework for Automatic Lung Cancer Detection from Lung CT Images.

[18] Kavitha BC, Naveen KB (2022). Image Acquisition and Pre-processing for Detection of Lung Cancer using Neural Network.

[19] Causey JL (2022). Spatial pyramid pooling with 3D convolution improves Lung Cancer Detection, in*IEEE/ACM transactions on Computational Biology and Bioinformatics*. 1 March-April.

[20] Ahmed I, Chehri A, Jeon G, Piccialli F (2023). Automated Pulmonary Nodule Classification and Detection Using Deep Learning Architecture. IEEE/ACM Trans Comput Biol Bioinform.

[21] Thakur A, Gupta M, Sinha DK, Mishra KK, Venkatesan VK, Guluwadi S (2024). Transformative breast Cancer diagnosis using CNNs with optimized ReduceLROnPlateau and Early stopping Enhancements. Int J Comput Intell Syst.

[22] Albalawi E, Thakur A, Ramakrishna MT, Khan B, Sankaranarayanan S, Almarri SB, Aldhyani T (2024). Oral squamous cell carcinoma detection using EfficientNet on histopathological images. Front Med..

[23] Shah AA, Malik HAM, Muhammad A, Alourani A, Butt ZA (2023). Deep learning ensemble 2D CNN approach towards the detection of lung cancer. Sci Rep.

[24] Alzubaidi MA, Otoom M, Jaradat H. Comprehensive and Comparative Global and Local Feature Extraction Framework for Lung Cancer Detection Using CT Scan Images, in *IEEE Access*. 2021;9:158140–54. 10.1109/ACCESS.2021.3129597.

[25] Mathio D, Johansen JS, Cristiano  S, Medina JE, Phallen J, Larsen KR, Velculescu E (2021). Detection and characterization of lung cancer using cell-free DNA fragmentomes. Nat Commu.

[26] Mehmood S et al. Malignancy Detection in Lung and Colon Histopathology Images Using Transfer Learning With Class Selective Image Processing, in *IEEE Access*, vol. 10, pp. 25657–25668, 2022, 10.1109/ACCESS.2022.3150924.

[27] Dritsas E, Trigka M (2022). Lung cancer risk prediction with machine learning models. Big Data Cogn Comput.

[28] Masud M, Sikder N, Nahid AA, Bairagi AK, AlZain MA (2021). A machine learning approach to diagnosing lung and colon cancer using a deep learning-based classification framework. Sensors.

[29] Naseer S, Akram T, Masood M, Rashid, Jaffar A. Lung Cancer Classification Using Modified U-Net Based Lobe Segmentation and Nodule Detection, in *IEEE Access*, vol. 11, pp. 60279–60291, 2023, 10.1109/ACCESS.2023.3285821.

[30] Bharathy S, Pavithra R (2022). Lung Cancer Detection using Machine Learning. In 2022 International Conference on Applied Artificial Intelligence and Computing (ICAAIC).

[31] Kasinathan G, Jayakumar S (2022). Cloud based lung tumor detection and stage classification using deep learning techniques. BioMed Res Int.

[32] Das S (2023). Automated Prediction of Lung Cancer Using Deep Learning Algorithms. Applied Artificial Intelligence.

[33] Tasnim N (2024). A Deep Learning Based Image Processing Technique for Early Lung Cancer Prediction. 2024 ASU International Conference in Emerging Technologies for Sustainability and Intelligent Systems (ICETSIS).

[34] Safta W (2024). Advancing pulmonary nodule diagnosis by integrating Engineered and Deep features extracted from CT scans. Algorithms.

[35] Khaliq K (2023). LCCNet: a deep learning based Method for the identification of lungs Cancer using CT scans. VFAST Trans Softw Eng.

[36] Nigudgi S (2023). Lung cancer CT image classification using hybrid-SVM transfer learning approach. Soft Comput.

[37] Diwakar M, Singh P, Shankar A (2021). Multi-modal medical image fusion framework using co-occurrence filter and local extrema in NSST domain. Biomed Signal Process Control.

[38] Das M, Gupta D, Bakde A (2024). An end-to-end content-aware generative adversarial network-based method for multimodal medical image fusion. Data Analytics Intell Sys.

[39] Jie Y, Xu Y, Li X, Tan H. (2024). TSJNet: A Multi-modality Target and Semantic Awareness Joint-driven Image Fusion Network. *arXiv preprint arXiv:2402.01212*.

[40] Dhaundiyal R, Tripathi A, Joshi K, Diwakar M, Singh P. Clustering based multi-modality medical image fusion. In: Journal of Physics: Conference Series. 2020 (Vol. 1478, No. 1, p. 012024). IOP Publishing.

[41] Diwakar M, Singh P, Shankar A, Nayak RS, Nayak J, Vimal S, Sisodia D (2022). Directive clustering contrast-based multi-modality medical image fusion for smart healthcare system. Netw Model Anal Health Inf Bioinf.

